# Paper-based potentiometric sensing devices modified with chemically reduced graphene oxide (CRGO) for trace level determination of pholcodine (opiate derivative drug)

**DOI:** 10.1039/d1ra00581b

**Published:** 2021-03-26

**Authors:** Hisham S. M. Abd-Rabboh, Abd El-Galil E. Amr, Elsayed A. Elsayed, Ahmed Y. A. Sayed, Ayman H. Kamel

**Affiliations:** Department of Chemistry, Faculty of Science, Ain Shams University Cairo 11566 Egypt ahkamel76@sci.asu.edu.eg +20-1000361328; Chemistry Department, Faculty of Science, King Khalid University Abha 61413 Saudi Arabia habdrabboh@kku.edu.sa; Pharmaceutical Chemistry Department, Drug Exploration & Development Chair (DEDC), College of Pharmacy, King Saud University Riyadh 11451 Saudi Arabia ahmedyahia009@gmail.com aamr@ksu.edu.sa +966-565-148-750; Applied Organic Chemistry Department, National Research Center Dokki 12622 Giza Egypt; Zoology Department, Faculty of Science, King Saud University Riyadh 11451 Saudi Arabia eaelsayed@ksu.edu.sa; Chemistry of Natural and Microbial Products Department, National Research Centre Dokki 12622 Cairo Egypt

## Abstract

Robust, reliable and cost-effective paper-based analytical device for potentiometric pholcodine (opiate derivative drug) ion sensing has been prepared and characterized. A printed pholcodinium (PHL)^2+^/5-nitrobarbiturate (NB)^−^ ion-association complex as a sensory material-based all-solid-state ion-selective electrode (ISE) on a chemically reduced graphene oxide (CRGO) solid-contact, and a printed all-solid-state Ag/AgCl reference electrode, has been combined on a hydrophobic paper substrate coated with fluorinated alkyl silane (CF_3_(CF_2_)_7_CH_2_CH_2_SiCl_3_, C^F^_10_). The sensors revealed a potentiometric slope of 28.7 ± 0.3 mV dec^−1^ (*R*^2^ = 0.9998) over a linear range starting from 2.0 × 10^−7^ M to 1.0 × 10^−2^ M and a detection limit of 0.04 μg mL^−1^. The repeatability and stability of the pholcodine paper-based sensor was found to be 2.32%. The RSD% (*n* = 6) was found to be 2.67% when using five different paper-based sensors. The sensor revealed an excellent selectivity towards PHL over dextromethorphan, codeine, ephedrine, carbinoxamine, caffeine, ketamine, and K^+^, Na^+^ and Ca^2+^ ions. It showed a good recovery (94–104%) for the determination of PHL in different artificial serum samples. The presented paper-based analytical device was successfully introduced for PHL determination in different pharmaceutical formulations (*i.e.* syrups and suspensions) containing pholcodine. The current work can be considered as a promising possible analytical tool to obtain cost-effective and disposable paper-based potentiometric sensing devices. These devices can be potentially manufacturable at large scales in pharmaceutical, clinical and forensic applications for opiate drug assessment.

## Abstract 2

Robust, reliable and cost-effective paper-based analytical device for potentiometric pholcodine (opiate derivative drug) ion sensing has been prepared and characterized.

## Graphical abstract

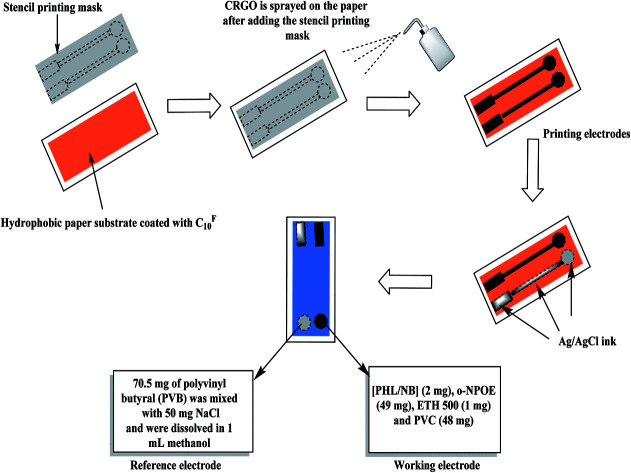

## 1. Introduction

Pholcodine (3-*O*-morpholinoethylmorphine), a semi-synthetic chemical compound derived from morphine, is used as an opioid cough suppressant (anti-tussive agent) with no analgesic or addictive properties and cold symptoms. Its comprehensive benefit/risk evaluation was recently revised by the European Medicines Agency (EMA).^1^ Most of the studies conducted between the 1960s and the 1980s proved its effectiveness in treating acute unproductive cough. But these studies showed weak methodology against the most recent comparative study, published in 2006. This study showed that the efficacy of both pholcodine and dextromethorphan was similar.^2^ Based on the available information for this drug, EMA recommended the maintaining marketing authorizations for products containing pholcodine.^1^ From the point of view of chemistry, pholcodine and Neuromuscular blocking agents (NMBAs) are believed to share the same immunoglobulin E (IgE)-binding epitope, which contains the quaternary ammonium ion (QAI) or its tertiary variety.^3^ Pholcodine has been shown to stimulate the production of IgE antibodies to QAI sensitization properties, and thus are an alternative source for NMBAs sensitization. In contrast to codeine, pholcodine is not metabolized into morphine in humans, a fact that may contribute to a more favorable toxicity profile: it is metabolized and eliminated much more slowly than codeine. So, it appears to be devoid of addiction liability in man.^4^

Different analytical methods were reported for the determination of pholcodine including non-aqueous titration,^5^ chemiluminescence,^4,6^ UV/Vis spectrophotometry,^7^ capillary gas chromatography (GC),^8,9^ GC-mass spectrometry (MS),^10,11^ high-performance liquid chromatography (HPLC),^12–16^ ultra-performance liquid chromatography (UPLC)-MS/MS,^17^ thin-layer chromatography,^18^ capillary electrophoresis (CE),^19^ electrochemistry^20^ and 1HNMR-pH titration.^21^

The use of electrochemical sensors and especially potentiometric sensors has become greatly expanded and now occupies a large part in different applications such as pharmaceutical, environmental and biomedical analysis.^22–33^ This type of sensors is fast, accurate, and cost-effective in addition to adequate detection and selectivity limits. Conventional sensors “liquid based sensors” encounter difficulties in miniaturization and have limited applications. Moreover, the lower detection limits were restricted by ion fluxes across the membrane with zero current.^31^ Solid contact electrodes eliminated the presence of internal filling solution. This type of sensors is characterized by their ease of miniaturization, convenient storage and maintenance, in addition to their minimum detection due to diminishing ion fluxes.^32,33^ The insertion of solid-contact transducing material minimizes or eliminate the ill-defined interface that is responsible for the potential-drift of the sensor.^34^

The direct way for enhancing the capacitance is to enhance the contact area between the membrane sensor and the core solid contact layer without necessarily increasing the geometric projection of the solid contact.^35^ This strategy can be demonstrated by using various nano-structured carbon materials with well-controlled structures and tunable surfaces. These carbon materials are characterized by their chemical stability and revealed high specific surface areas due to their nano-structures.

Recently, paper-based analytical devices have a great attention as a powerful platform for field and point-of-care (POC) testing. This is attributed to their self-pumping ability and utility for many different analytical measurements.^36^ These types of devices when designed for electrochemical detection using small and portable electronics, the sensitivity and selectivity of the paper-devices can be improved over visual colorimetric detection without sacrificing portability. The importance of use paper in analytical applications lies in its characteristics of low-cost, thin, lightweight, flexible, compatible with a wide array of patterning methods, easily disposable, and generation of flow without external pumps. In the past decade, there is a significant growth of academic research on paper-based analytical tools has been seen. All of these academic-research papers explored the capability of this planar-platforms to perform sensitive and selective analytical testing that is routinely carried out using bench-top instruments.^37,38^

In this work, we report for the first time a novel potentiometric solid-contact sensor for the determination of pholcodine as an opioid cough suppressant. Reduced graphene oxide (CRGO) was used as a solid contact ion-to electron transducer. The membrane sensor includes the ion-association complex of pholcodine (PHL) and 5-nitrobarbiturate (NB) in *o*-nitrophenyloctyl ether (*o*-NPOE) as a solvent mediator. The electrochemical performance characteristics of the presented sensors were evaluated. Advantages of these sensors include design simplicity, fast response, cost-effectiveness, adequate precision, high accuracy, high-analytical throughput, good response-stability, low detection limit and high selectivity over many common interfering ions. The proposed sensors are introduced for PHL determination in different commercially available cough pent syrup.

## 2. Materials and methods

### 2.1. Chemicals and reagents

Chemically reduced graphene oxide (CRGO), tetradodecylammonium tetrakis (4-chlorophenyl) borate (ETH500), 2-morpholin-4-ium-4-ylethanesulfonate (MES), *o*-nitrophenyl octyl ether (*o*-NPOE), 5-nitrobarbiturate (NB), fluorinated alkyl silane (CF_3_(CF_2_)_7_CH_2_CH_2_SiCl_3_, C^F^_10_) and high molecular weight polyvinyl chloride (PVC) were obtained from Sigma-Aldrich (St. Lois, MO, USA). Ag/AgCl ink (E2414) was purchased from Ercon (Wareham, MA). Tetrahydrofuran (THF) and dimethyl formamide (DMF) were purchased from Fluka. Pholcodine (PHL) was kindly supplied by Amoun Pharmaceutical Co. (El-Obour City, Cairo, Egypt) with a purity assay > 99.0%.

All chemical reagents were prepared using de-ionized water (18.2 MΩ cm, Millipore Milli-Q system). MES-buffer solution (50 mM, pH 5.0) was used as a working buffer solution. A 10^−2^ M stock solution of pholcodine was prepared after dissolving its corresponding amount in 0.1 M HCl solution and completed to the mark of 50 mL measuring flask with the working buffer to assist the pH of the solution at pH 5. The working standard solutions (10^−8^ to 10^−3^ M) were prepared from the stock solution daily and prior to the measurements by appropriate dilution with MES, pH 5.

An artificial serum solution was prepared as described elsewhere.^39^ In brief, 6.8 g of NaCl was mixed with 2.2 g NaHCO_3_, 0.4 g KCl, 0.2 g CaCl_2_, 0.1 g MgSO_4_, 0.12 g Na_2_HPO_4_ and 0.02 g NaH_2_PO_4_. All were dissolved in 1.0 L de-ionized water.

### 2.2. Instrumentations

All mV measurements were carried out using benchtop mV/pH meter (PXSJ-216, INESA Scientific Instrument Co., Ltd, Shangahi, China). The reversed-current chronopotentiometry was carried out using Metrohm potentiostat/galvanostat (Autolab, model 204, Herisau, Switzerland). Three-electrode cell was used for these measurements. Pt wire and Ag/AgCl/1 M (KCl) were used as auxiliary and reference electrodes, respectively.

### 2.3. Electrode design and membrane preparation

**Fig. 1:**
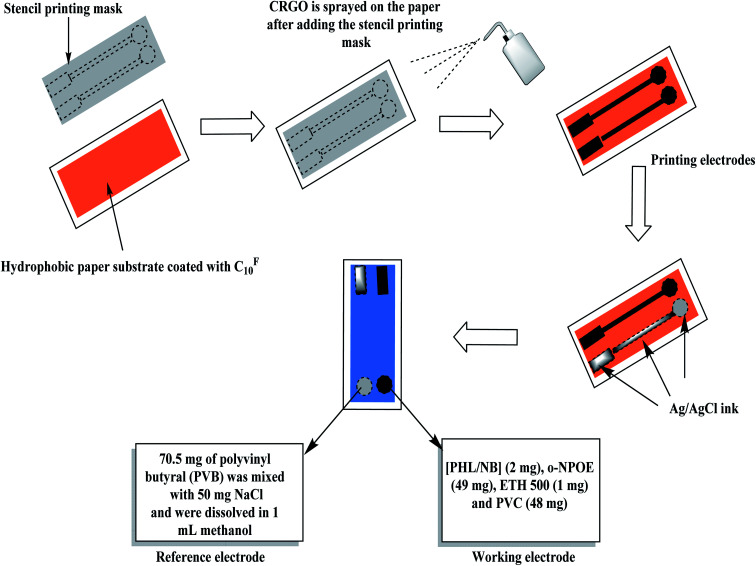
A schematic representation of the fabrication of paper-based ISE.

The electrode substrate was made from a hydrophobic paper. The paper substrate was mixed with 20 μL C^F^_10_ and then were placed in a Petri-dish. The C^F^_10_ was evaporated in a vacuum drying chamber at 80 °C for 30 min. A uniform layer of C^F^_10_ is then coated on the paper-electrode substrate.^40^ A 5 mL of CRGO was dispersed ultrasonically in ethanol for 1 h. The solution was than sprayed onto the modified C^F^_10_-paper through a stainless-steel mask. The solution of the sensing membrane was prepared by dissolving 100 mg of the components in 1.5 mL tetrahydrofuran as: [PHL][NB]_2_ (2 mg), *o*-NPOE (49 mg), ETH 500 (1 mg) and PVC (48 mg). A 20 μL of the membrane cocktail was drop-casted over the modified C^F^_10_-paper and left to dry for 2 h to produce the presented the modified PHL-ISE. For constructing the solid-state reference electrode, Ag/AgCl ink was printed on the C^F^_10_-paper with an orifice width 2 mm. Consequently, 70.5 mg of polyvinyl butyral (PVB) was mixed with 50 mg NaCl and were dissolved in 1 mL methanol. This cocktail solution was used as a reference membrane solution for the paper-based reference electrode. A schematic representation of the fabrication of paper-based ISE was shown as Fig. 1.

### 2.4. Electrochemical characterization

All potentiometric measurements were carried out at room temperature. All-solid-state electrodes were inserted in a 9 mL MES buffer at pH 5. Aliquots (0.1–1.0 mL) of 10^−5^ to 10^−2^ M standard PHL solution was introduced and the potential change was recorded after each PHL addition. A calibration-plot was then constructed after plotting the potential *versus* logarithm of PHL concentration. The plot was then used for PHL assessment in real samples. ES measurements were carried out under the open circuit potential with a frequency range from 0.1 Hz to 100 KHz, and the AC amplitude was 50 mV. These tests were performed to evaluate the ion-to-electron transducer performance. For CP measurements, reverse currents (±1 nA) were applied to evaluate the short-term potential stability of the presented electrode.

### 2.5. Sample application

To test the applicability of the proposed sensors, recovery of PHL from an artificial serum solution was evaluated. 1 mL of the prepared artificial serum solution was mixed with 9 mL of the MES buffer and then introduced to 25 mL beaker. Different aliquots of PHL standard solution were mixed with the serum solution. The paper-based electrochemical cell was then inserted to the solution and the potential after reaching the equilibrium response was then recorded. The amount of PHL spiked was calculated using the constructed calibration plot. The applicability of the presented paper-based sensors was tested on commercial pharmaceutical preparations as real samples. Syrups (Cyrinol, Apic Pharm. Co., Egypt; labeled 4 mg PHL mL^−1^) and suspensions (Marynol, Glaxo Wellcome, Egypt; labeled 4 mg mL^−1^) were purchased from local pharmacies. Aliquot (10 mL) of either the syrup or suspension sample was accurately transferred into a 50 mL volumetric flask and mixed with 1 mL of 0.1 M HCl, then the solution was completed to the mark with MES-buffer solution to adjust the pH of the solution at pH 5. 10 mL of the solution was transferred to a 25 mL beaker and the electrochemical cell was then inserted in the solution. The potential after reaching equilibrium is then recorded and the amount of PHL was calculated from the constructed calibration plot.

## 3. Results and discussion

### 3.1. Paper-based sensor fabrication

**Fig. 2:**
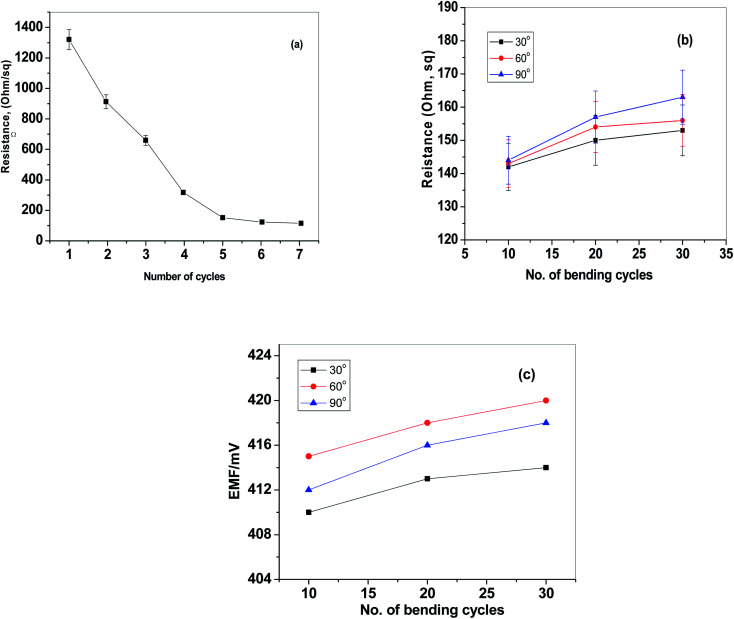
(a) The relationship between the conductivity and the number of print cycles of CRGO; effect of cycles of bending at different bending angles on (b) resistance and (c) EMF.

The design and fabrication of the paper-based sensor was presented and shown in Fig. 1. The paper surface was treated with C^F^_10_ to enhance its hydrophobicity and to eliminate the water-layer effect. After, the paper was modified with a layer of reduced graphene oxide layer as an ion-to electron transducing material. As shown in Fig. 2a, the electrical resistance of the modified paper was recorded as a function of the number of spraying cycles. As the number of spraying cycles increases, the resistance of the paper decreases. The resistance remains constant and reaches ∼145 Ω after seven spraying cycles. To test the mechanical flexibility of the paper-based sensor, the paper was bent several times with different angle of bending (*i.e.* 30°, 60°, and 90°). The drift recorded in the resistance and electromotive force (EMF) was ∼40 Ω and 3.2 mV, respectively (Fig. 2b and c). These results indicate that the prepared paper-based sensor revealed good mechanical flexibility and high conductivity.

### 3.2. Potentiometric performance of the paper-based sensor

**Fig. 3:**
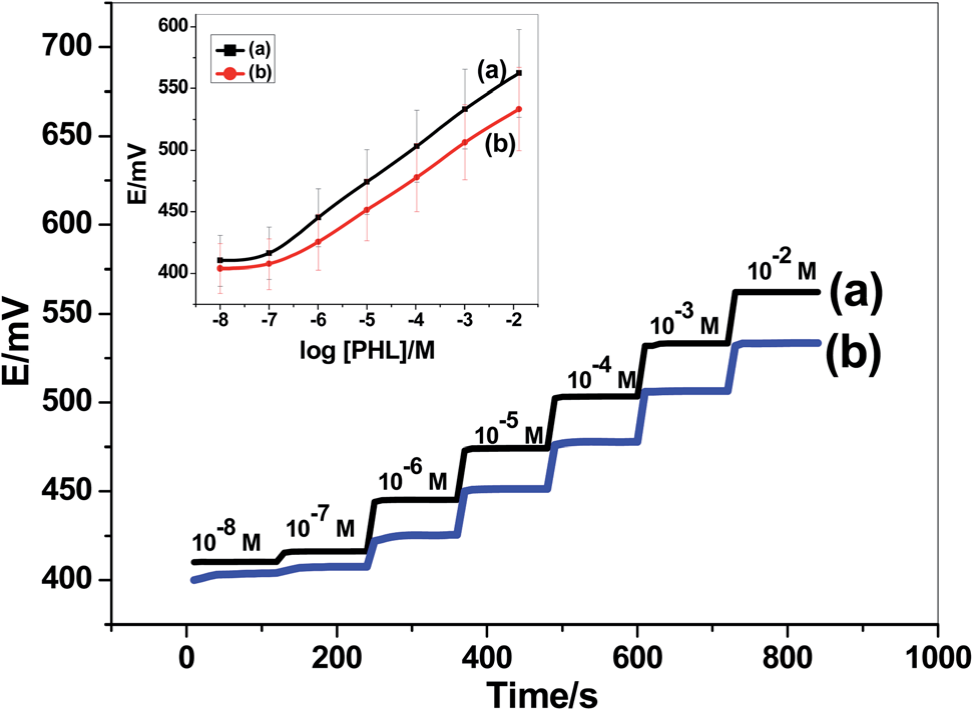
Calibration plots of the presented PHL sensors: (a) paper/CRGO/PHL-ISE and (b) GC/PHL-ISE.

The performance characteristics of the presented PHL paper-based sensor (*i.e.* paper/CRGO/PHL-ISE) were evaluated after measuring the potential response at different PHL concentrations (10^−8^ M to 10^−2^ M). The calibration plot and time trace of the presented sensor was shown in Fig. 3. The presented paper-based sensor revealed a Nernstian slope of 28.7 ± 0.3 mV dec^−1^ (*R*^2^ = 0.9998) over a linear range starting from 2.0 × 10^−7^ M to 1.0 × 10^−2^ M. The detection limit was calculated according to IUPAC guidelines^41^ and found to be 0.04 μg mL^−1^. For comparison, the potentiometric performance of the sensor but without the transducing layer (*i.e.* CRGO) was also evaluated on a glassy-carbon electrode (GC/PHL-ISE). The sensor revealed a potentiometric slope of 26.5 ± 0.7 mV dec^−1^ (*R*^2^ = 0.9997) over the linear range 5.0 × 10^−7^ to 1.0 × 10^−2^ M and detection limit of 0.12 μg mL^−1^. The slope was less than the slope obtained in case of the presence of CRGO transducer. In addition, a potential drift is also noticed specially in low concentrations of PHL. These results confirmed the necessity of inserting the CRGO layer as an ion-to electron transducer before the ion-sensing membrane.

The repeatability and stability of pholcodine paper-based sensor were investigated using the proposed potentiometric measurements of 10.0 μM PHL. After six measurements, the relative standard deviation (RSD%) was found to be 2.32%. This can be considered as an adequate repeatability. The RSD% (*n* = 6) was found to be 2.67% when using five different paper-based sensors. This revealed a good reproducibility for the prepared sensors. Storing the sensors for 4 weeks retained 95% of their initial response. All of these results indicated a good stability, reproducibility and long life-span for the presented modified paper-based sensors, and can be used successfully for pholcodine determination.

Life-span of the presented paper-based sensors is very important to be checked for routine analysis. Therefore, the performance characteristics of these electrodes were examined day-to-day by performing daily calibration. The slope and detection limit were found to be constants over three working-days. After the fourth day to the tenth day, both calibration slope and detection limit start to decrease. After one-week working, a complete failure was noticed. Therefore, all performance characteristics of the proposed paper-based analytical device such as detection limit, response time, linear range and calibration slope were found to be reproducible within their original values over a period of at least one week.

Influence of the pH on the potential response of the presented sensor was tested for (1 × 10^−4^ and 1 × 10^−3^ M) PHL solutions. It can be seen that the electrodes exhibited a higher and stable potential over the pH range 4.2–6.0. At pH < 4, there is an observed potential drift which may be attributed to an interference from H^+^ ions. On the other hand, the potential decreased at pH values > 9.0. This can be explained on the basis of non-ionized pholcodine formation (p*K*_a_ = 9.3).^42^ Over the pH range 6.5–8.5, a mixture between mono-and di-valent pholcodine is formed.^21^ All subsequent measurements were carried out at pH 5 using 50 mM MES-buffer solution.

The selectivity behavior of the proposed sensor was evaluated and selectivity coefficient (*K*^Pot^_i,j_) values were calculated using the method presented by Bakker [*i.e.* modified separate solution method (MSSM)].^43^ The selectivity values for the sensor over common organic and inorganic cations were presented in Table 1. The sensor revealed an excellent selectivity towards PHL over dextromethorphan, codeine, ephedrine, carbinoxamine, caffeine, ketamine, K^+^, Na^+^ and Ca^2+^ ions. An interference is noticed from morphine and ethyl morphine. The results obtained reflected a good selectivity for the presented paper-based sensor and offered a great potential for trace-level monitoring of PHL in different matrices.

**Table 1:** The selectivity coefficients (log *K*^Pot^_PHL,B_) of C/PEDOT:PSS/pholcodine-ISE^a^

Interfering ion, B	log *K*^Pot^_PHL,B_ ± SD
Morphine	+1.3 ± 0.3
Ethylmorphine	+0.7 ± 0.6
Ephedrine	−3.2 ± 0.2
Codeine	−3.3 ± 0.1
Dextromethorphan	−3.8 ± 0.2
Carbinoxamine	−4.2 ± 0.7
Caffeine	−4.3 ± 0.2
Ketamine	−3.4 ± 0.3
K^+^	−4.8 ± 0.2
Ca^2+^	−5.1 ± 0.1
Na^+^	−4.9 ± 0.3

a ±Standard deviation of three measurements.

### 3.3. Electrochemical measurements

**Fig. 4:**
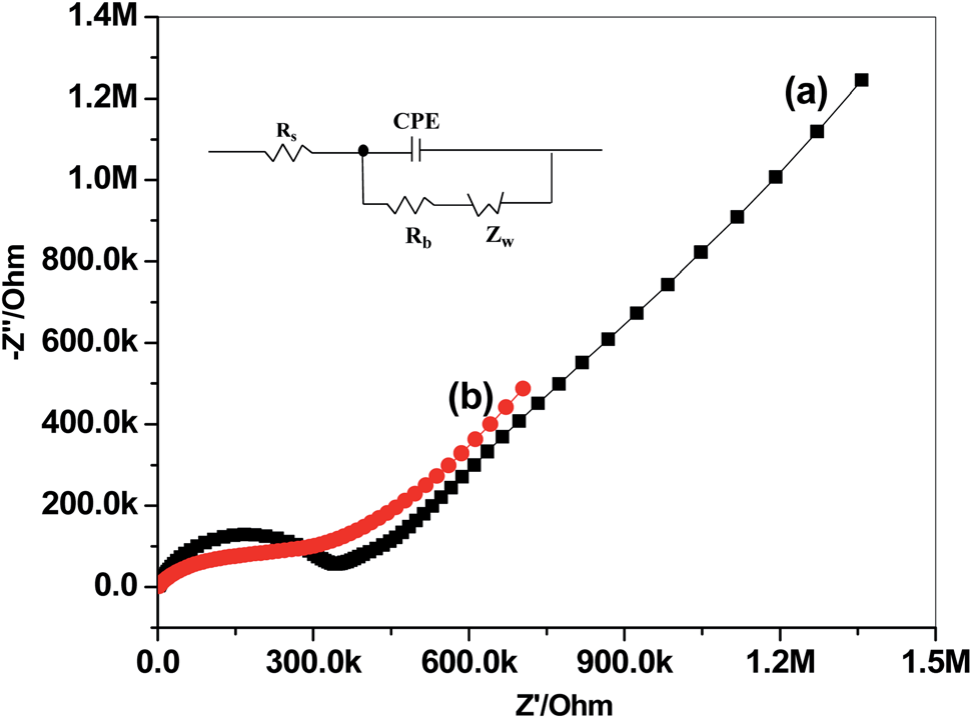
Impedance spectra of (a) GC/PHL-ISE and (b) paper/CRGO/PHL-ISE.

Impedance spectroscopic measurements for paper/CRGO/PHL-ISE and GC/PHL-ISE were carried out in 10 mM PHL and the spectra were shown in Fig. 4. Both sensors displayed high-frequency circles, which are related to bulk resistance of the ion-sensing membrane.^44^ The equivalent circuit is composed of the resistance of the solution (*R*_s_), an interfacial constant phase element (CPE), the bulk membrane resistance (*R*_b_) and the Warburg diffusion element (*Z*_w_) (Inset, Fig. 4). As shown in the impedance spectra, the *R*_b_ values were found to be 0.33 MΩ and 0.28 MΩ for GC/PHL-ISE and paper/CRGO/PHL-ISE, respectively. This suggests that CRGO layer serves as transducer enhances the ion-to-electron charge transfer.

**Fig. 5:**
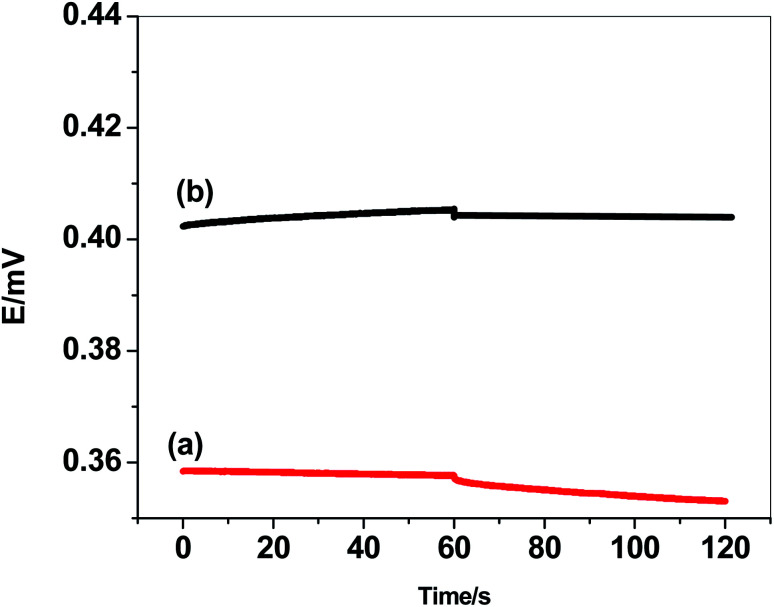
Current reversal chronopotentiometry for (a) GC/PHL-ISE and (b) paper/CRGO/PHL-ISE.

Current-reversal chronopotentiometric measurements were carried out for the evaluation of the capacitance and short-term potential stability of the prepared sensors (Fig. 5). In these type of measurements, cathodic and anodic current (1 nA) was applied on the prepared sensor for 60 s and the potential values were recorded.^45^ The potential drifts (Δ*E*/Δ*t*) were found to be is 103.5 and is 39.2 μV s^−1^ for GC/PHL-ISE and paper/CRGO/PHL-ISE, respectively. In addition, the interfacial double-layer capacitance [*C* = *I*/(Δ*E*/Δ*t*)] was calculated for both GC/PHL-ISE and paper/CRGO/PHL-ISE and found to be 9.6 and 25.5 μF, respectively.

**Fig. 6:**
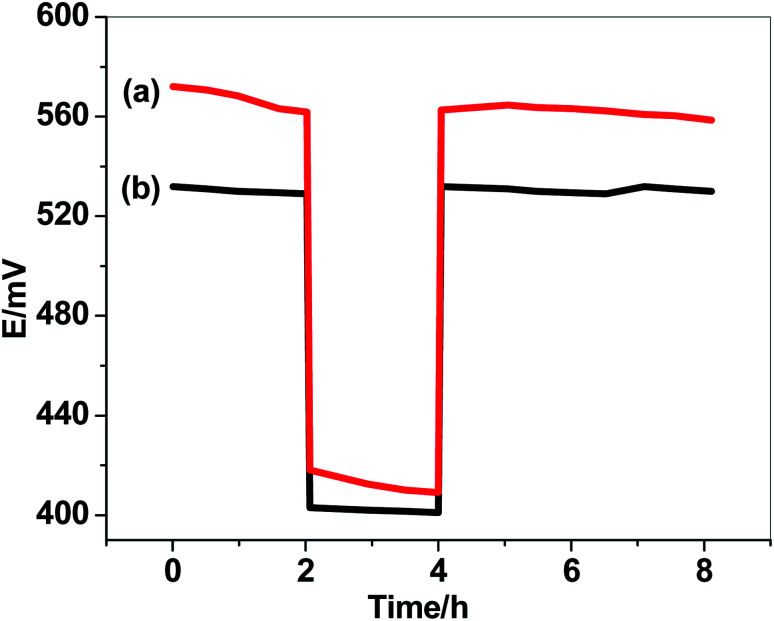
Water-layer test for (a) GC/PHL-ISE and (b) paper/CRGO/PHL-ISE.

The formation of water-layer between the ion-sensing membrane and the electrode substrate significantly effects on the electrode potential-stability. Water-layer test were performed and the results were shown in Fig. 6. The potentials of either GC/PHL-ISE or paper/CRGO/PHL-ISE were measured after inserting the sensors for 2 hours in 10 mM PHL solution; then they moved into 0.1 M NaCl solution four 2 hours, and finally returned back to 10 mM PHL solution for several hours. The results showed a continuous potential drift for GC/PHL-ISE while a potential stability is observed for paper/CRGO/PHL-ISE. This high potential-stability observed in paper/CRGO/PHL-ISE reflects the super-hydrophobic property of CRGO which eliminate the formation of water-layer. These results were further achieved by a long-term study of potential-drift which showed a small positive drift of 0.2 mV h^−1^ over 12 hours as a test period.

### 3.4. Analytical applications

The presented sensors were successfully applied for PHL determination in spiked artificial serum samples. Following the procedure, five samples were analyzed containing PHL concentration on the therapeutic range. The same samples were also measured with a standard method suggested by the British Pharmacopeia for comparison.^46^ The results were accurate when comparing the data obtained by the presented potentiometric method with those obtained by the reference standard method (Table 2). No significant difference between the values at 95% confidence was noticed.

**Table 2:** Determination of PHL in spiked artificial serum samples^a^

Sample	Amount spiked, μM	Amount found, μM ± SD*				*F*-test
		Potentiometry	Recovery, %	Reference method,^46^	Recovery, %	
1	1.0	0.94 ± 0.04	94.0	0.97 ± 0.01	97	3.21
2	2.5	2.6 ± 0.3	104.0	2.48 ± 0.2	99.2	2.45
3	5.0	4.73 ± 0.4	94.6	5.02 ± 0.3	100.4	4.14
4	10.0	9.65 ± 0.5	96.5	9.92 ± 0.1	99.2	3.34
5	50.0	47.4 ± 1.4	94.8	50.3 ± 0.2	100.6	4.42

a ±SD* (standard deviation for average of 5 measurements).

The suggested potentiometric method was also introduced for PHL determination different pharmaceutical formulations containing pholcodine. Syrups (Cyrinol, Apic Pharm. Co., Egypt; labeled 4 mg PHL mL^−1^) and suspensions (Marynol, Glaxo Wellcome, Egypt; labeled 4 mg mL^−1^) were purchased from local pharmacies. The obtained results were compared with those of the official British Pharmacopoeia (BP) and shown in Table 3. The data obtained confirmed the validity of using the presented sensors for the routine determination of PHL in pharmaceutical formulations.

**Table 3:** Determination of PHL in different pharmaceutical formulation samples^a^

Sample	Labeled amount, mg mL^−1^	Amount found, mg mL^−1^ ± SD*				*F*-test
		Potentiometry	Recovery, %	Reference method,^46^	Recovery, %	
Cyrinol, Apic Pharm. Co., Egypt (syrup)	4.0	3.91 ± 0.2	97.7	3.97 ± 0.1	99.2	2.32
Marynol, Glaxo Wellcome, Egypt (suspension)	4.0	4.14 ± 0.4	103.5	3.88 ± 0.5	97.0	2.45

a ±SD* (standard deviation for average of 5 measurements).

## 4. Conclusions

In this work, we aimed to develop reliable, robust, cost-effective paper-based potentiometric analytical devices that are suitable future mass-production for pholcodine (*i.e.*, opiate derivative drug) determination. An all-solid-state ISE for PHL ion in conjunction with a reference Ag/AgCl electrode were successfully designed on a common filter-paper. The sensory material was the ion association complex between pholcodinium ion and 5-nitrobarbaturate. It was dispersed in a PVC membrane plasticized with *o*-NPOE. Chemically reduced graphene oxide (CRGO) was used as a solid-contact transducer. The sensors revealed a potentiometric slope of a of 28.7 ± 0.3 mV dec^−1^ (*R*^2^ = 0.9998) over a linear range starting from 2.0 × 10^−7^ M to 1.0 × 10^−2^ M and a detection limit of 0.04 μg mL^−1^. The presented paper-based potentiometric analytical device revealed good repeatability, reproducibility and stability. It exhibited good recovery range (*i.e.* 94–104%) of PHL from artificial serum samples. In addition, the device was successfully applied for rapid quantification of PHL in syrup and suspension pharmaceutical samples collected from the local market. The presented work can be adapted to further low-cost and disposable paper-based potentiometric sensing devices produced at large scales with high-speed and reproducible paper-printing technology.

## Author contributions

The listed authors contributed to this work as described in the following: H. S. M. A.-R. and A. H. K. gave the concepts of the work, interpretation of the results, the experimental part and prepared the manuscript, A. H. K., H. S. M. A.-R. and A. E.-G. E. A. cooperated in the preparation of the manuscript and A. H. K. and H. S. M. A.-R. performed the revision before submission. E. A. E. revealed the financial support for the work. All authors have read and agreed to the published version of the manuscript.

## Conflicts of interest

The authors declare that there are no conflicts of interest. All authors have approved the manuscript and agree with the submission to your esteemed journal.

## Supplementary Material
